# Commentary: Metabolomics-Based Studies Assessing Exercise-Induced Alterations of the Human Metabolome: A Systematic Review

**DOI:** 10.3389/fphys.2020.00353

**Published:** 2020-04-22

**Authors:** Alex Castro, Renata Garbellini Duft, Ana Carolina de Mattos Zeri, Claudia Regina Cavaglieri, Mara Patrícia Traina Chacon-Mikahil

**Affiliations:** ^1^Laboratory of Exercise Physiology, School of Physical Education, University of Campinas, Campinas, Brazil; ^2^Brazilian Synchrotron Light Laboratory, Brazilian Center for Research in Energy, Campinas, Brazil

**Keywords:** metabolism, metabolomics, exercise, sports, experimental design

We have read with interest the study of Sakaguchi et al. (2019), which proposes a qualitative appraisal of recent metabolomics-based studies (published over the past decade) exploring exercise-induced alterations of the human metabolome. The authors devised a scoring system ranging from zero (poor quality = below 4) to 11 (excellent quality = above nine) to attribute quality levels for each assessed study. The criteria used were based on research design (number of participants and study characteristics), methodology (analytical methods and statistical choices), and novelty (Sakaguchi et al., 2019). Although this systematic review was indeed well-conducted, some concerns need to be addressed, particularly on the validity of the scoring system used.

First, for an appropriate sample size (N) for metabolomics and exercise studies, the authors attributed two points to studies with N > 20 or N > 13 for parallel and crossover designs, respectively, or zero if they presented a smaller sample size (Sakaguchi et al., 2019). However, no calculation of statistical power was presented to support these suggested numbers. To improve the reproducibility of future investigations on this topic, well-established methodological principles should not be overlooked, and sample size should be based on statistical power analysis (Krzywinski and Altman, 2013). Ensuring that sample sizes are large enough to detect the effects of interest is an essential part of study design, especially in “omics” studies, where multiple outcomes are tested, and a large number of true positive results may be missed due to insufficient statistical power (van Iterson et al., 2009; Krzywinski and Altman, 2013).

Secondly, for study characterization, the authors suggest that metabolomics investigations should use a randomized controlled design, along with more than two-timepoint data collection and/or a duration of over 3 weeks (chronic studies only) (Sakaguchi et al., 2019). We do subscribe to the view that studies using randomized controlled trials should be encouraged, since they are the most rigorous way to evaluate the cause-effect relation between treatment and outcome (Sibbald and Roland, 1998; Concato et al., 2000). However, we would like to point out that the number of data collection points and the number of weeks in longitudinal studies (chronic response) is highly dependent on the experimental design and research objectives and should not be used as a criterion for disqualifying a study. For instance, for longitudinal studies with parallel randomized control groups with samples obtained only at rest, two timepoints of data collection (pre- and post-intervention) are sufficient to assess exercise-induced alterations in the basal human metabolome (Huffman et al., 2014; Glynn et al., 2015; Duft et al., 2017; Brennan et al., 2018). For cross-over studies (acute response), we suggest that a control session (no exercise) be included in the experimental design for a clearer interpretation of the effects of exercise compared to those of prolonged fasting (Shrestha et al., 2015; Karimpour et al., 2016; Li-Gao et al., 2019). The inclusion of any additional data collection timepoints after exercise sessions would depend on the research goals.

Thirdly, regarding analytical methods, the authors assigned a different score to the LC-MS/MS, GC-MS, and 1H NMR methods, which suggests a hierarchy of importance between them (Sakaguchi et al., 2019). However, they failed to provide a clear account of the reasoning behind this decision. In our view, it is not appropriate to make a hierarchical quality comparison between metabolomics platforms, as they are complementary, and there is no single technique that is capable of quantifying all the chemical compounds in a given sample at the same time. Therefore, the choice of analytical methods should be supported by the specific objectives of each study. For example, if the objective of the study includes the investigation of metabolites with polar characteristics, NMR may be a sound choice, whereas if the compounds of interest are hydrophobic or are in low concentrations, GC-MS would be a better alternative. The study carried out by Karimpour et al. (2016) has shown an interesting approach to comparing these three platforms in the identification of compounds in human plasma (Karimpour et al., 2016).

Fourthly, regarding statistical support, the authors attribute a gradual increase in the score to the addition of factors in the analysis and the application of multivariate/bioinformatic statistical methods, when compared to traditional univariate statistical analysis (Sakaguchi et al., 2019). Although the use of multivariate statistical methods and bioinformatics has driven new discoveries in metabolomics due to their high capacity to extract relevant information from large data sets (Johnson et al., 2015; Meier et al., 2017), the choice of these tools depends on the experimental design or on the type of research question, and as such, they do not necessarily ensure an improved study quality. Therefore, we suggest that regardless of the statistical approach taken, the reader should question whether the underlying assumptions have been carefully addressed. For example, a partial least squares discriminative analysis (PLS-DA), used for supervised group classification (Worley and Powers, 2013; Ren et al., 2015), requires validation parameters, which is often difficult to achieve due to the small sample size and large number of variables common in human metabolomics studies (Antonelli et al., 2019). In this case, the quality of the study should be linked not only to the mere use of the PLS-DA model but also to whether appropriate validation is presented for it, including cross-validation tests. This would allow for a proper examination of the magnitude of the values of *R*^2^ (goodness of model fit) and *Q*^2^ (model predictive capacity), as well as the discrepancy between them (model overfitting), permutation tests (statistical significance of the classification model), and the application of corrections for multiple tests in subsequent univariate analyzes, among other relevant parameters, to support the findings of the study (Westerhuis et al., 2008; Triba et al., 2015).

Fifthly, the authors point out as a quality criterion of the publication the addition of new information to the literature (novelty). Such a statement should be preceded by a retrospective analysis of the literature published up to the time of publication of each article included in the review. It is not productive to disqualify a past scientific paper without considering the available scientific base and accumulated knowledge. Acknowledging limitations and advances provided by previous studies has enabled the development of research in this emerging field of metabolomics and exercise.

Other important points not mentioned by Sakaguchi et al. (2019) deserve some comments, as they may also provide direction for future investigations and contribute to achieving comparable metabolomics results between studies.

- Standardization of participant's preparation prior to collection of biological samples at rest and pre-exercise, since postprandial time, diet composition, and time after the previous training session are likely to affect the metabolome (Daskalaki et al., 2015; Shrestha et al., 2015; Karimpour et al., 2016; Giskeødegård et al., 2019). In this sense, we suggest the collection of biological samples at rest after 10–12 h overnight fasting or 90–120 min after a standardized meal previous to an exercise session, which is expected to present reasonable stabilization of postprandial metabolism (Shrestha et al., 2015; Karimpour et al., 2016; Giskeødegård et al., 2019; Li-Gao et al., 2019).
- Presentation of the reliability of measurements (between and/or intra-experiments) for each metabolite so that the reader may evaluate the true magnitude of intervention effects in relation to measurement errors as demonstrated by few recent studies (Berton et al., 2016; Wang et al., 2018; Castro et al., 2019; Giskeødegård et al., 2019; Li-Gao et al., 2019).
- Presentation of the obtained spectra, when possible, accompanied by the identification of the spectral peaks corresponding to each metabolite found, in order to enable the replicability.
- Individualized exercise prescription, based on physiological thresholds whenever possible, to accurately address the individual metabolic characteristics for a more reliable comparison of metabolic adaptations between and within individuals (Wasserman, 1986; Garber et al., 2011; Riebe et al., 2018; Weatherwax et al., 2019).

Finally, we suggest an open debate among experts in the fields of mass spectrometry, NMR, exercise physiology, and statistics to bring us closer to a consensus on standardization guidelines such as has been undertaken by previous initiatives (Lindon et al., 2005; Beckonert et al., 2007; Sansone et al., 2007; Emwas et al., 2015; Spicer et al., 2017). This broader discussion may be more effective in improving the quality and robustness of further experiments in the emerging field of metabolomics and exercise than the limited qualification of studies already conducted using a score built from unconsolidated criteria.

## Author Contributions

AC, RD, AZ, CC, and MC-M have fully reviewed and criticized the original article. AC drafted the first version of the commentary. RD, CC, AZ, and MC-M contributed to the revision and editing of the manuscript for important intellectual content. All authors reviewed and approved the final manuscript.

## Conflict of Interest

The authors declare that the research was conducted in the absence of any commercial or financial relationships that could be construed as a potential conflict of interest.
